# Supplementary material for: Multivariate genomic architecture of cortical thickness and surface area at multiple levels of analysis
Source: Nat Commun. 2023 Feb 20;14:946. doi: 10.1038/s41467-023-36605-x (PMC9941500; doi:10.1038/s41467-023-36605-x)
Supplement: Supplementary file 1 — Supplementary Information [file 41467_2023_36605_MOESM1_ESM.pdf]

Supplementary Information

Multivariate Genomic Architecture of Cortical Thickness and Surface Area at Multiple Levels of Analysis

Table of Contents

Correlated Factors Model Results 2

Supplementary Figure 1. Correlated Factor Model Results for Cortical Thickness. Figure 4

Supplementary Figure 2. Correlated Factor Model Results for Surface Area. 5

Supplementary Figure 3. Genetic Heatmap for Left Hemisphere Cortical Thickness from UK Biobank. 6

Supplementary Figure 4. Genetic Heatmap for Right Hemisphere Cortical Thickness from UK Biobank. 7

Supplementary Figure 5. Genetic heatmap for Left Hemisphere Surface Area from UK Biobank. 8

Supplementary Figure 6. Genetic heatmap for Right Hemisphere Surface Area from UK Biobank. 9

Supplementary Figure 7. Biologically-derived features of the cortex that vary across the genomic factors of cortical thickness (CT). 10

Supplementary Figure 9a. Functionally-derived features of the cortex that vary across the genomic factors of cortical thickness (CT). 12

Supplementary Figure 9b. Functionally-derived features of the cortex that vary across the genomic factors of cortical thickness (CT). 13

Supplementary Figure 9c. Functionally-derived features of the cortex that vary across the genomic factors of cortical thickness (CT). 14

Supplementary Figure 10a. Functionally-derived features of the cortex that vary across the genomic factors of surface area (SA) 15

Supplementary Figure 10b. Functionally-derived features of the cortex that vary across the genomic factors of surface area (SA) 16

Supplementary Figure 11. Functional Enrichment for Genomic Factors in Bifactor Model 17

Supplementary Figure 12. Stratified Genomic SEM Results: Correlated and Bifactor Models. 18

.... 19

Supplementary Figure 13. UK Biobank Participant Sample Quality Control. 19

Online Supplement References 20

Correlated Factors Model Results

We present results for the bifactor model in the main text but consider results for the correlated factors model here (Supplementary Tables 2 and 3 for model results; Supplementary Figures 1-2 for path diagrams). On average, the five factors for each metric were highly genetically correlated (average factor r_g = .80 for CT; average r_g = .82 for SA), with some factors evincing more distinct genetic underpinnings (e.g., r_g = .66 across the CT cingulate [$F2_{CT}$] and occipital [$F4_{CT}$] factors). In addition, excluding the variation explained by the general factor, there was a 65.8% reduction for CT and 75.9% reduction for SA in the variation explained by the five factors for the bifactor model relative to the correlated factors model.

Enrichment. For CT, 9 annotations were found to be significantly enriched for at least one of the factors from the correlated factors model (Supplementary Table 10). This included: significant enrichment of the H3K27ac cingulate gyrus histone mark, endothelial cells, and PI genes for $F1_{CT}$; oligodendrocytes and the H3K4me1 cingulate gryus histone mark for $F2_{CT}$; and the middle hippocampus H3K27ac histone mark and PI genes for $F3_{CT}$. We identified 13 significant annotations for SA in the correlated factors model (Supplementary Figure 12; Supplementary Table 11). This included significant enrichment of endothelial cells and the H3K27ac histone mark within the anterior caudate for $F1_{SA}$; the intersection of microglia and PI genes for $F2_{SA}$; and the H3K4me3 histone mark in the dorsolateral prefrontal cortex and H3K27ac histone mark in the angular gyrus for $F3_{SA}$.

It can also be informative to consider how the patterns of enrichment identified here diverge from enrichment identified by ENIGMA for their global metrics of CT and SA.[1] For example, endothelial cells were highly enriched for global CT in ENIGMA, but were found to be uniquely enriched for the factor defined by central regions in the current analyses ($F1_{CT}$). Similarly, microglia were significant for global SA in ENIGMA, but only for $F2_{SA}$ in the current analyses. These findings tentatively indicate that prior enrichment findings for the global signal may be collapsing across patterns of enrichment unique to subclusters of contiguous brain regions. In some cases, annotations enriched at the factor level mapped onto the regions that defined the factor. For example, we observe that the cingulate factor ($F2_{CT}$) for CT was enriched for the tissue specific expression of the H3K4me1 histone mark in the cingulate gyrus.

g-factor. Results for SA revealed significant genetic correlations between all five factors and the *g*-factor in the context of the correlated factors model (average r_g = .22; range = .16-.25; Supplementary Table 12). There were no significant factor correlations for CT in the correlated factors model after correcting for multiple comparisons. However, we observe significant overlap across the residual genetic variance for VNR (also termed fluid intelligence in UKB) and the precentral region for the correlated factors model (partial r_g = .31, p = 1.02E-4). While this latter result should be reexamined as sample sizes continue to grow, it is consistent with prior findings from a semi-independent sample.[2]

Psychiatric. Results for CT revealed no significant correlations between any of the brain and psychiatric factors in the correlated factors model (Supplementary Table 14). For SA, a significant, negative genetic correlation was observed between the Neurodevelopmental disorders factor and $F2_{SA}$, defined by temporal and frontal regions (r_g = -.22, *SE* = .05, p = 2.43E-5). While this correlation was not significant in the bifactor model, it is of note that the

Neurodevelopmental factor, relative to the other psychiatric factors, evinced a pattern of generally stronger, negative associations with the other brain factors. Consistent with this observation, the Neurodevelopmental factor displayed the strongest genetic correlation with the general SA factor in the bifactor model (r_g = -.17, *SE* = .05, *p* = 1.19E-3), though this was not significant at a Bonferroni corrected threshold. Given the stringent Bonferroni correction applied here this should be reevaluated as larger samples become available.

Four nominally significant residual relationships were also identified between ASD and specific brain regions, the strongest of which was with the rostral anterior cingulate for the correlated factor (partial r_g = .180, *SE* = .051, *p* = 4.06E-4). Perturbations in the cingulate region more broadly have also been observed for prior phenotypic imaging and ASD studies.[3,4] The current findings are then informative for interpreting this prior literature given high levels of genetic and phenotypic overlap across psychiatric and structural imaging phenotypes. That is, the multivariate genomic analyses performed here indicate that ASD-cingulate associations can be considered as highly specific to these two phenotypes, as opposed to an indirect proxy for far more generalized pathways across highly correlated psychiatric and structural outcomes.

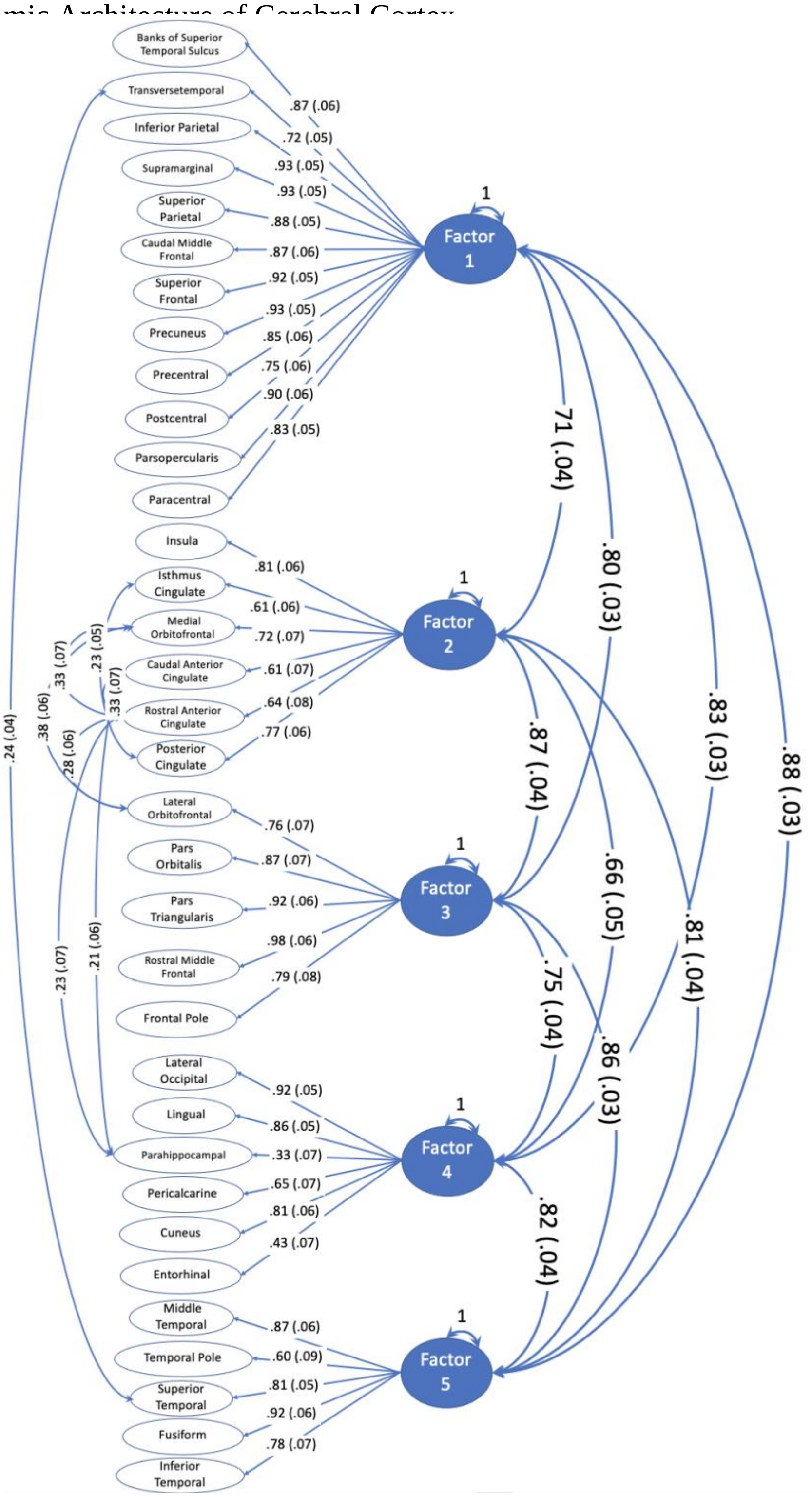

Supplementary Figure 1. Correlated Factor Model Results for Cortical Thickness. Figure depicts the standardized loadings from the five-factor correlated factor model results for cortical thickness. All models used unit variance identification (i.e., factor variances were fixed to 1) and the associations between factors consequently depict genetic correlations. The residual associations across pairs of brain regions depict residual genetic correlations that are standardized relative to their SNP-based heritabilities (i.e., not standardized with respect to the residual genetic variance). The residual variances of the brain region indicators are not depicted for simplicity, but can be found in Supplementary Table 2.

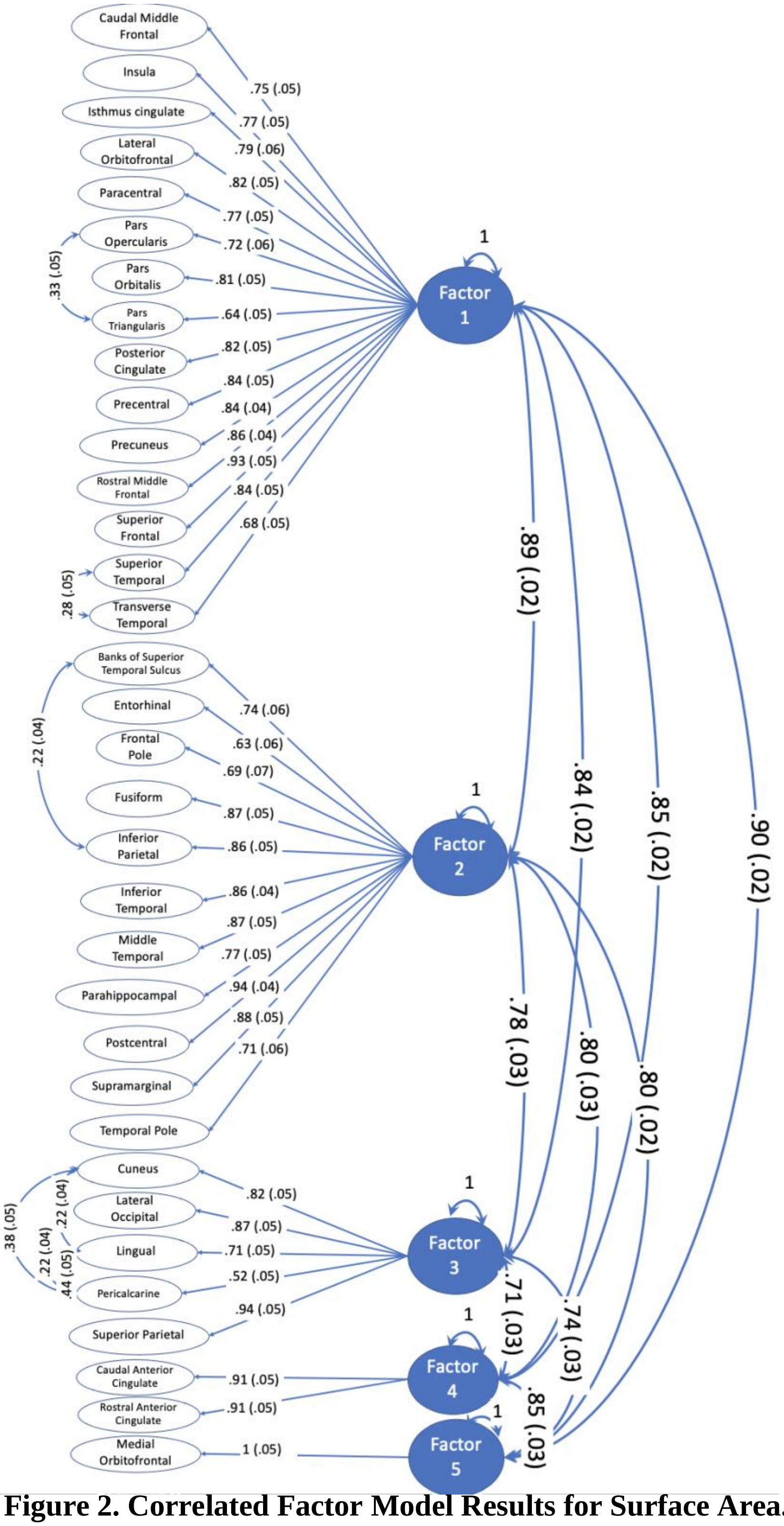

Supplementary Figure 2. Correlated Factor Model Results for Surface Area. Figure depicts the standardized loadings from the five-factor correlated factor model results for Surface Area. All models used unit variance identification (i.e., factor variances were fixed to 1) and the associations between factors consequently depict genetic correlations. The bivariate associations between individual brain regions depict residual genetic correlations that are standardized relative to their SNP-based heritabilities (i.e., not standardized with respect to the residual genetic variance). The residual variances of the brain region indicators are not depicted for simplicity, but can be found in Supplementary Table 3.

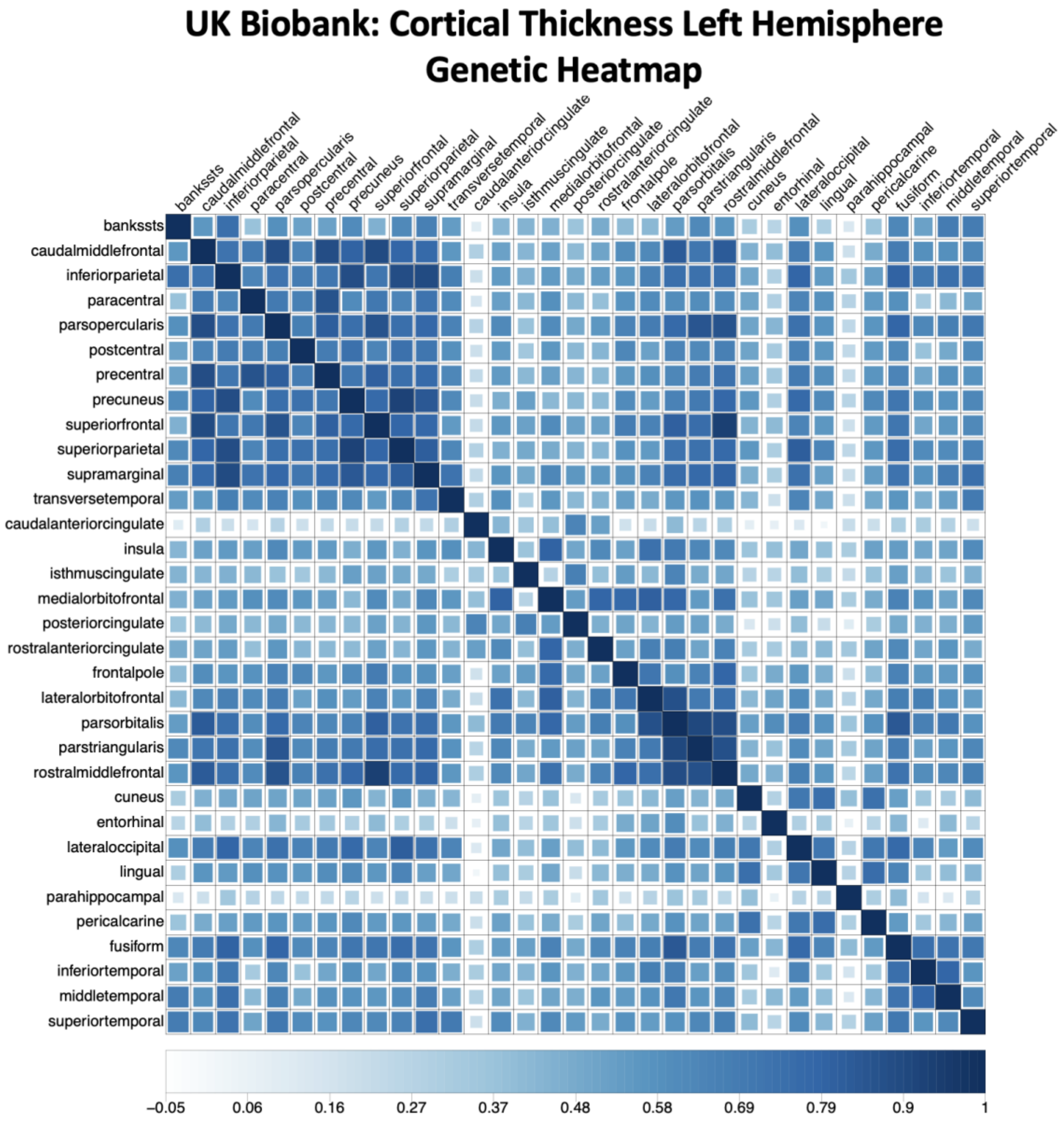

Supplementary Figure 3. Genetic Heatmap for Left Hemisphere Cortical Thickness from UK Biobank. Figure depicts the genetic heatmap, as estimated using LD-score regression, for cortical thickness in the left hemisphere from UK Biobank. The heatmap is ordered with respect to the ENIGMA cortical thickness factor model identified in Genomic SEM.

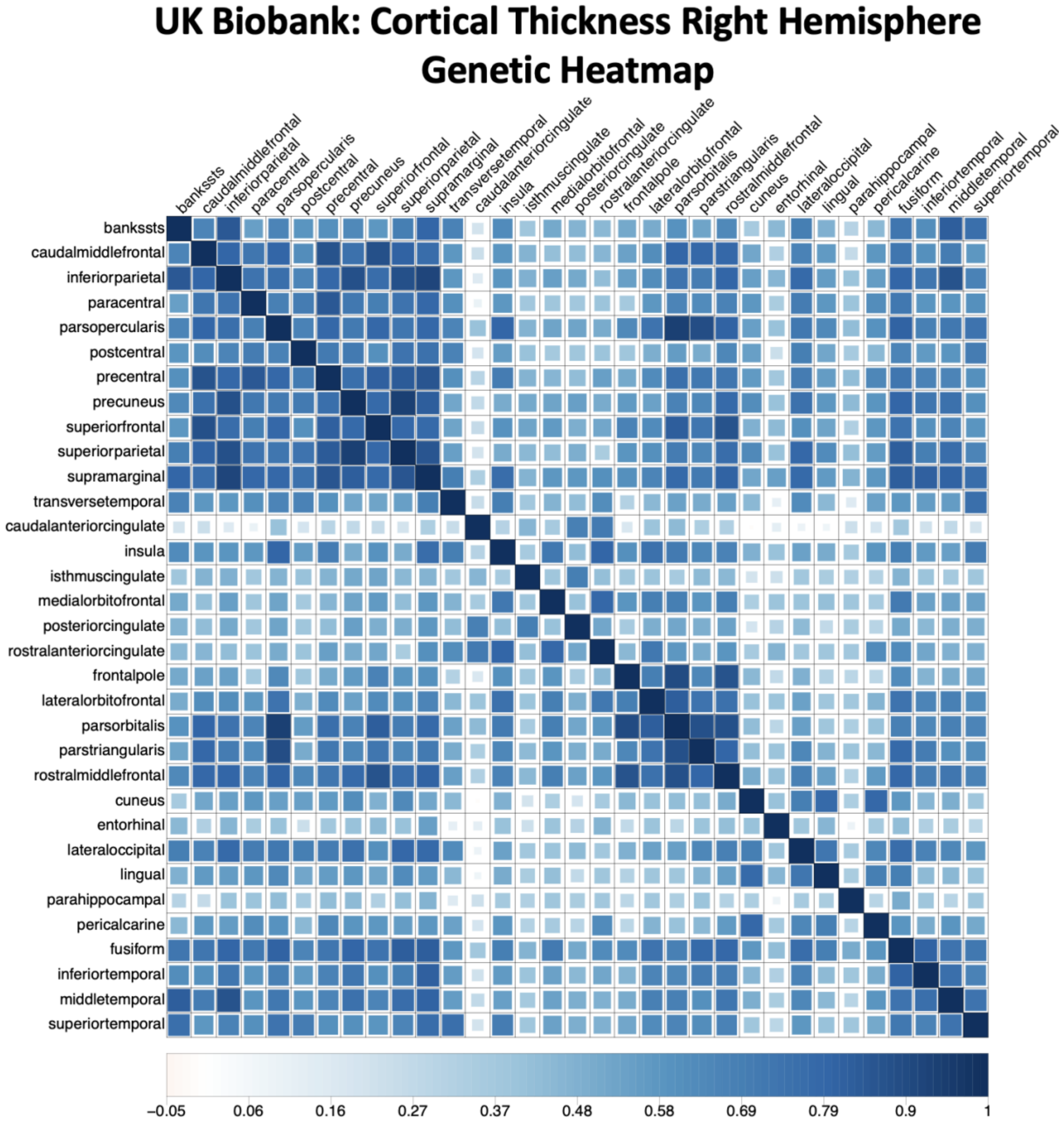

Supplementary Figure 4. Genetic Heatmap for Right Hemisphere Cortical Thickness from UK Biobank. Figure depicts the genetic heatmap, as estimated using LD-score regression, for cortical thickness in the right hemisphere from UK Biobank. The heatmap is ordered with respect to the ENIGMA cortical thickness factor model identified in Genomic SEM.

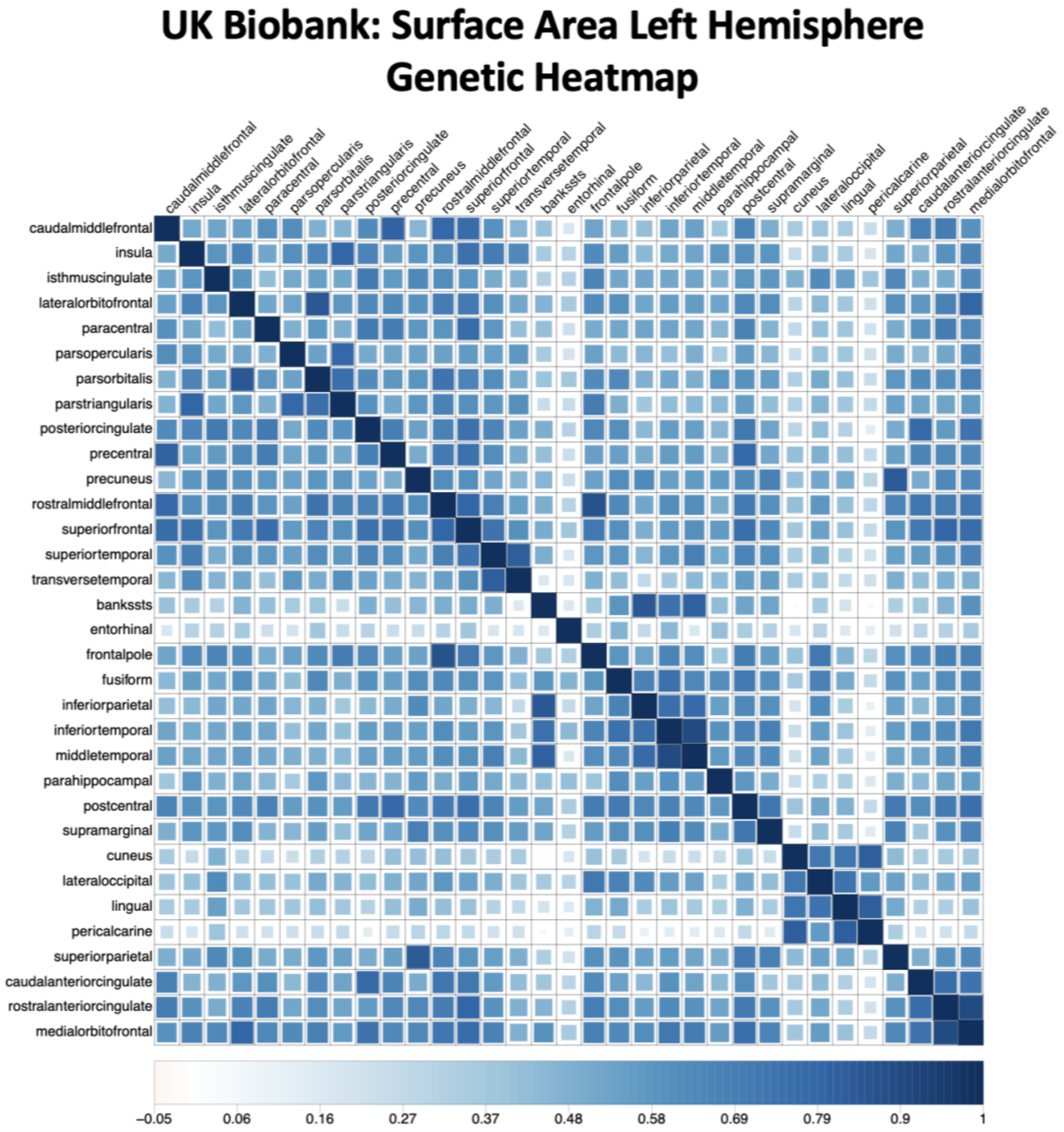

Supplementary Figure 5. Genetic heatmap for Left Hemisphere Surface Area from UK Biobank. Figure depicts the genetic heatmap, as estimated using LD-score regression, for surface area in the left hemisphere from UK Biobank. The heatmap is ordered with respect to the ENIGMA surface area factor model identified in Genomic SEM.

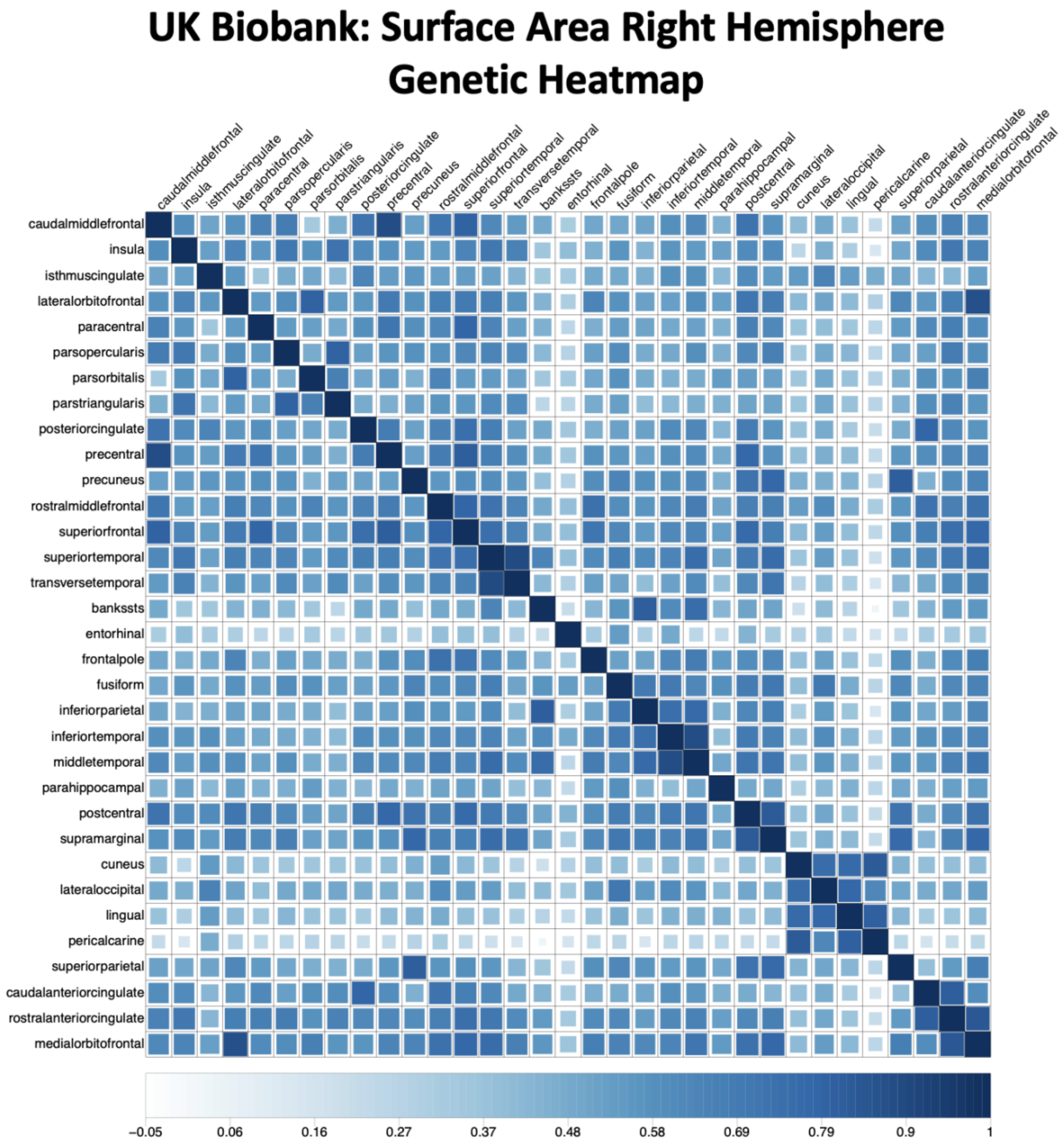

Supplementary Figure 6. Genetic heatmap for Right Hemisphere Surface Area from UK Biobank. Figure depicts the genetic heatmap, as estimated using LD-score regression, for surface area in the right hemisphere from UK Biobank. The heatmap is ordered with respect to the ENIGMA surface area factor model identified in Genomic SEM.

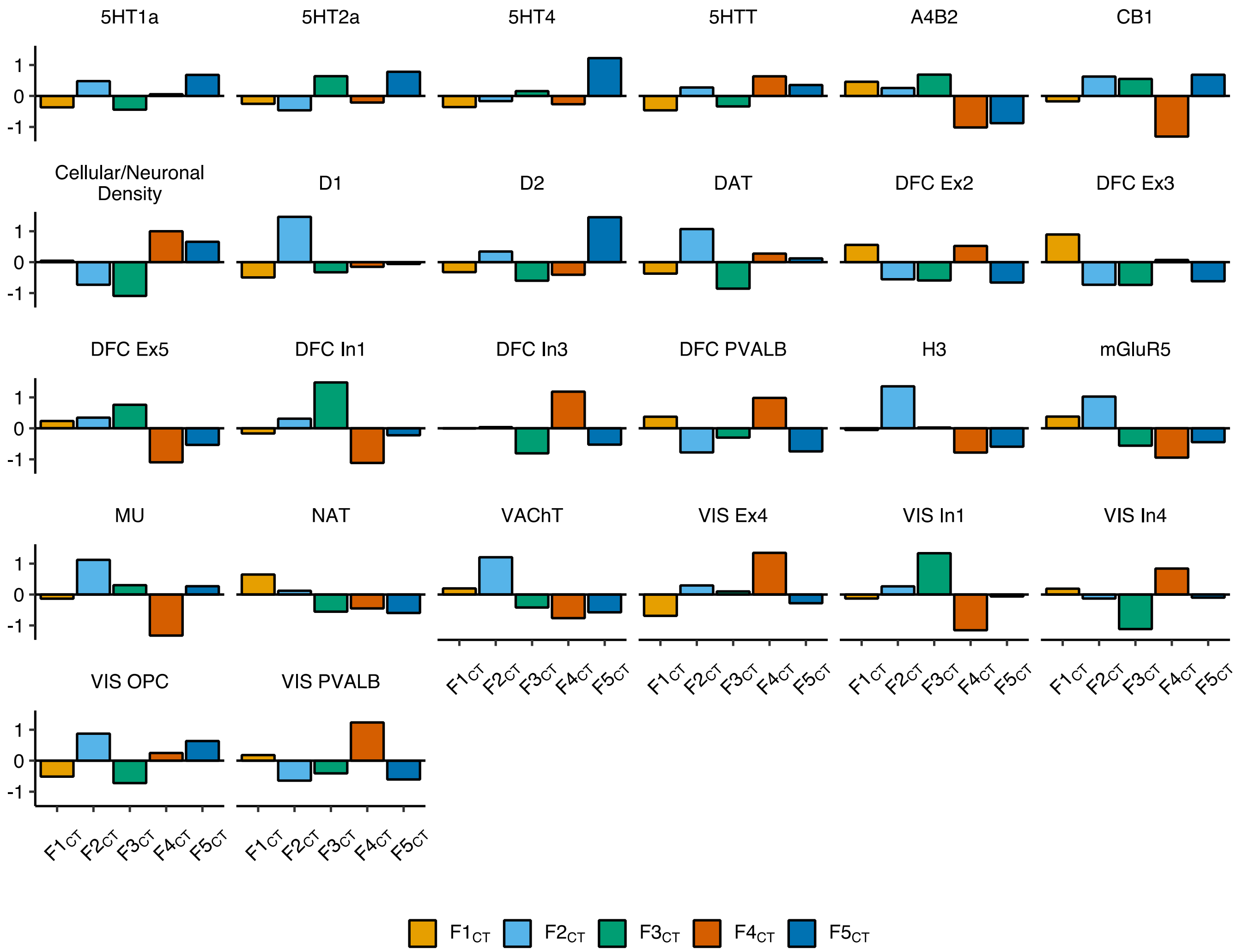

Supplementary Figure 7. Biologically-derived features of the cortex that vary across the genomic factors of cortical thickness (CT). Faceted bar charts of mean standardized values per factor for biologically-derived features of the cortex. All displayed features were significantly different across the five genomic factors of CT ($F1_{CT}$ to $F5_{CT}$) in an omnibus test of differences (FDR-corrected $P_{spin} < .05$; **Methods**). Full results are reported in Supplementary Table 9.

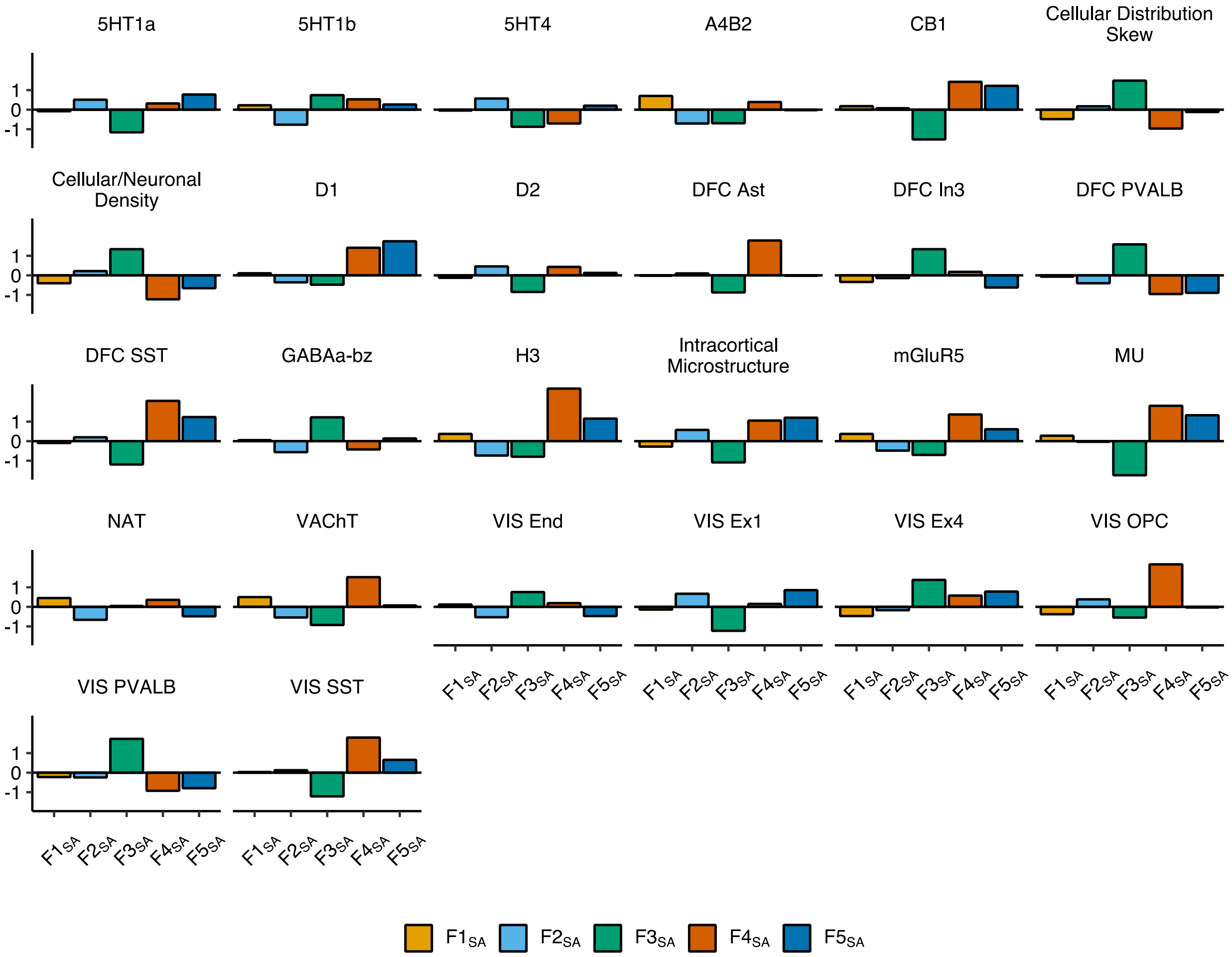

Supplementary Figure 8. Biologically-derived features of the cortex that vary across the genomic factors of surface area (SA). Faceted bar charts of mean standardized values per factor for biologically-derived features of the cortex. All displayed features were significantly different across the five genomic factors of SA ($F1_{SA}$ to $F5_{SA}$) in an omnibus test of differences (FDR-corrected $P_{spin} < .05$; **Methods**). Full results are reported in Supplementary Table 9.

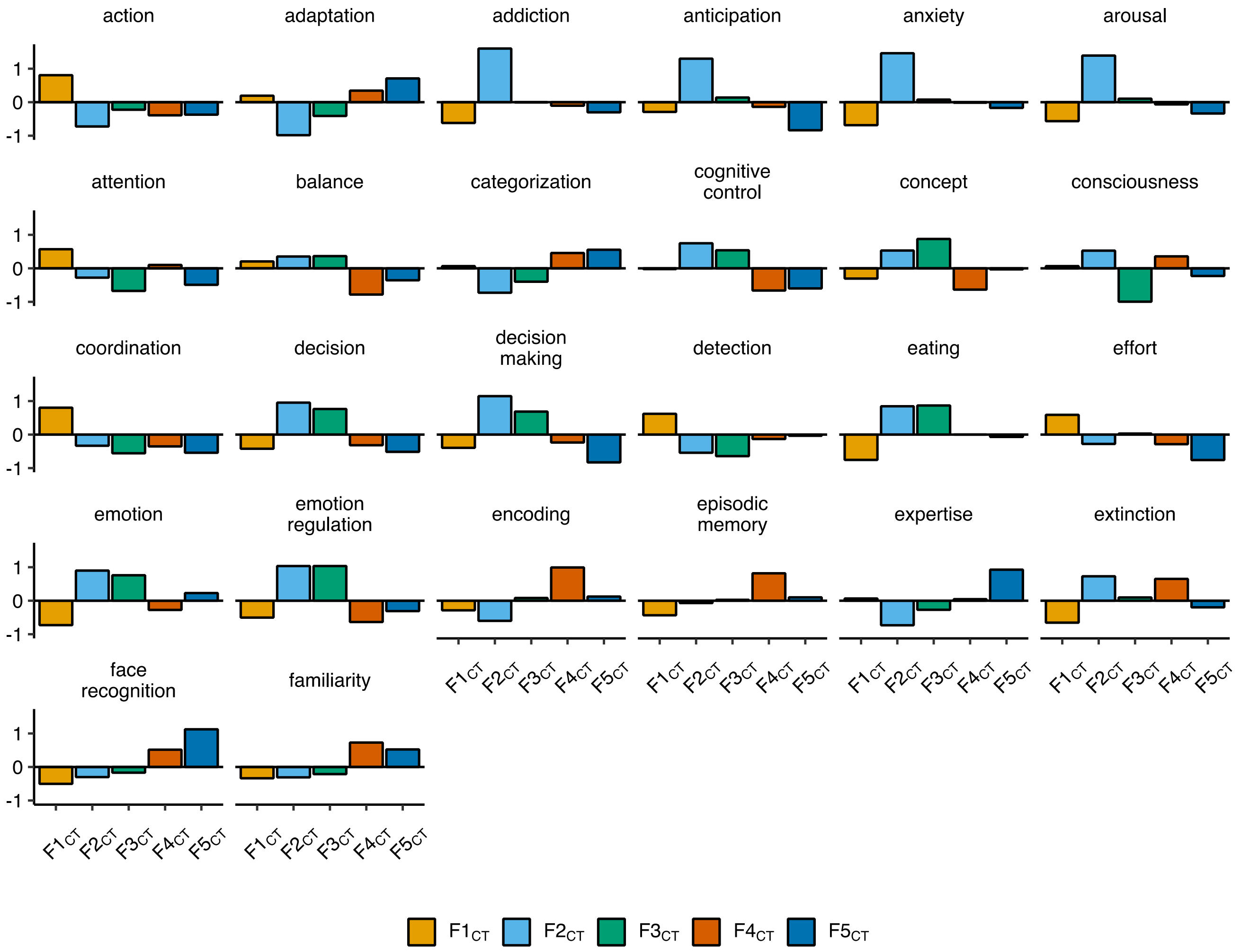

Supplementary Figure 9a. Functionally-derived features of the cortex that vary across the genomic factors of cortical thickness (CT). Faceted bar charts of mean standardized values per factor for functionally-derived features of the cortex. All displayed features were significantly different across the five genomic factors of CT ($F1_{CT}$ to $F5_{CT}$) in an omnibus test of differences (FDR-corrected $P_{spin} < .05$; **Methods**). Full results are reported in Supplementary Table 9.

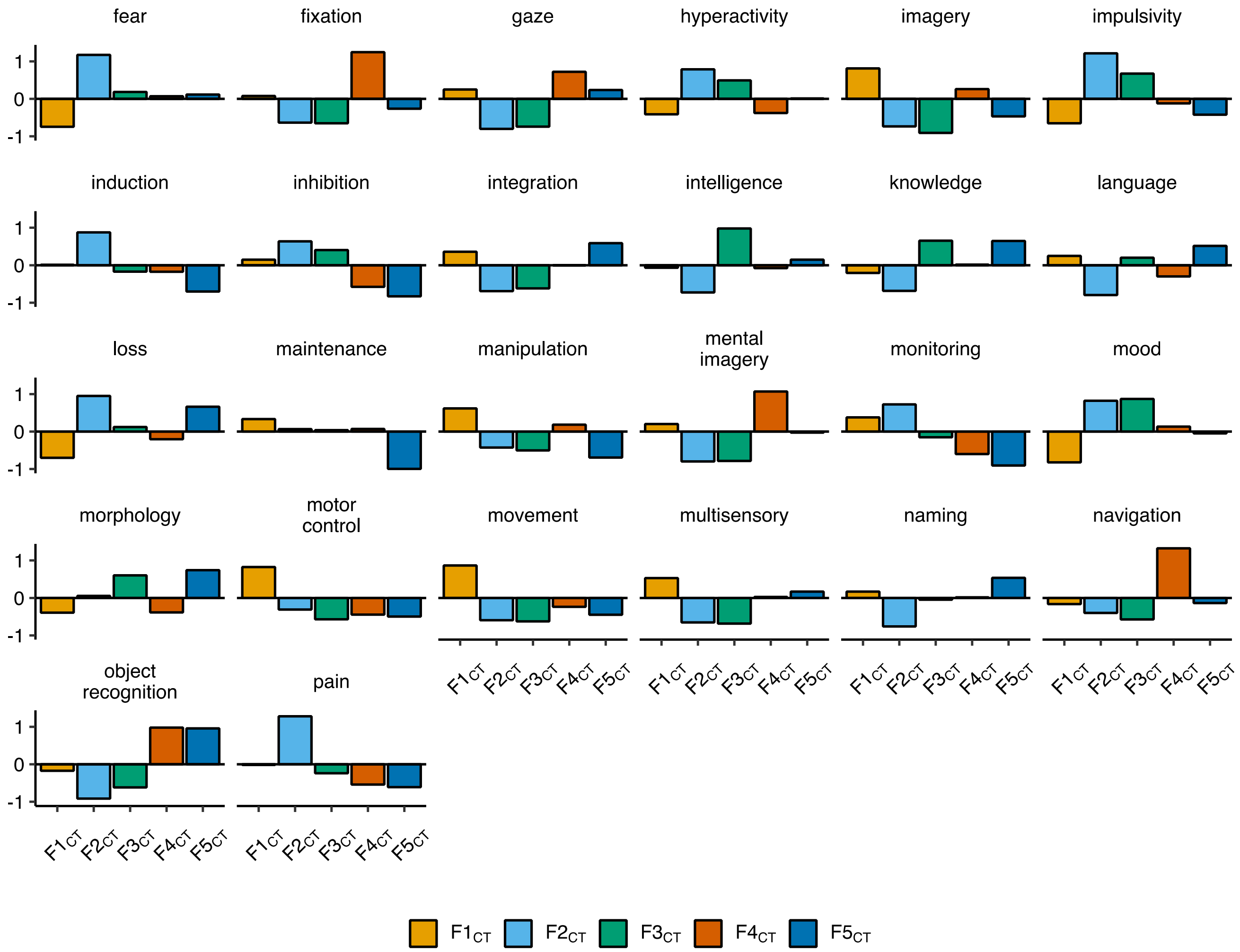

Supplementary Figure 9b. Functionally-derived features of the cortex that vary across the genomic factors of cortical thickness (CT). Faceted bar charts of mean standardized values per factor for functionally-derived features of the cortex. All displayed features were significantly different across the five genomic factors of CT ($F1_{CT}$ to $F5_{CT}$) in an omnibus test of differences (FDR-corrected P_{spin} < .05; **Methods**). Full results are reported in Supplementary Table 9.

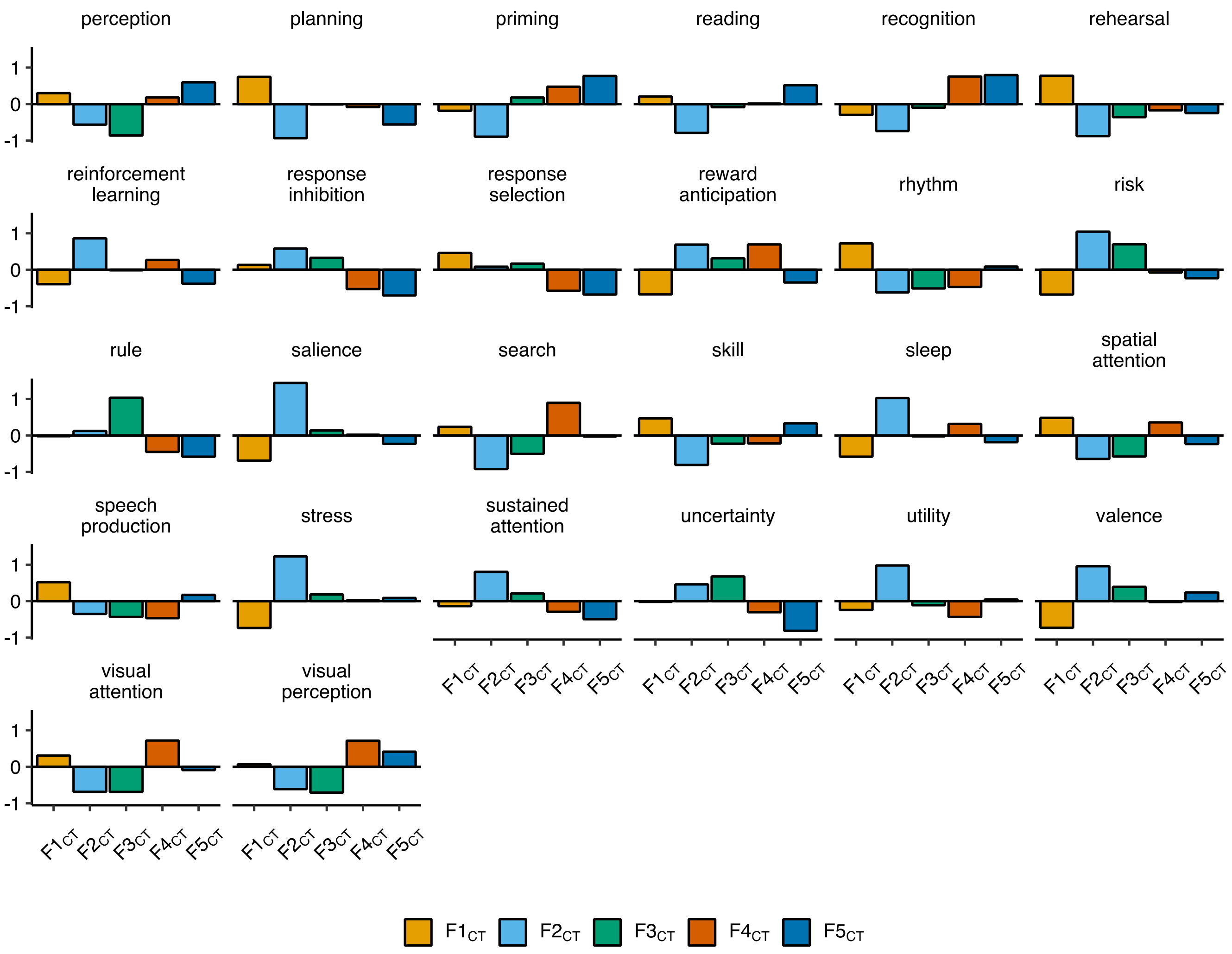

Supplementary Figure 9c. Functionally-derived features of the cortex that vary across the genomic factors of cortical thickness (CT). Faceted bar charts of mean standardized values per factor for functionally-derived features of the cortex. All displayed features were significantly different across the five genomic factors of CT ($F1_{CT}$ to $F5_{CT}$) in an omnibus test of differences (FDR-corrected $P_{spin} < .05$; **Methods**). Full results are reported in Supplementary Table 9.

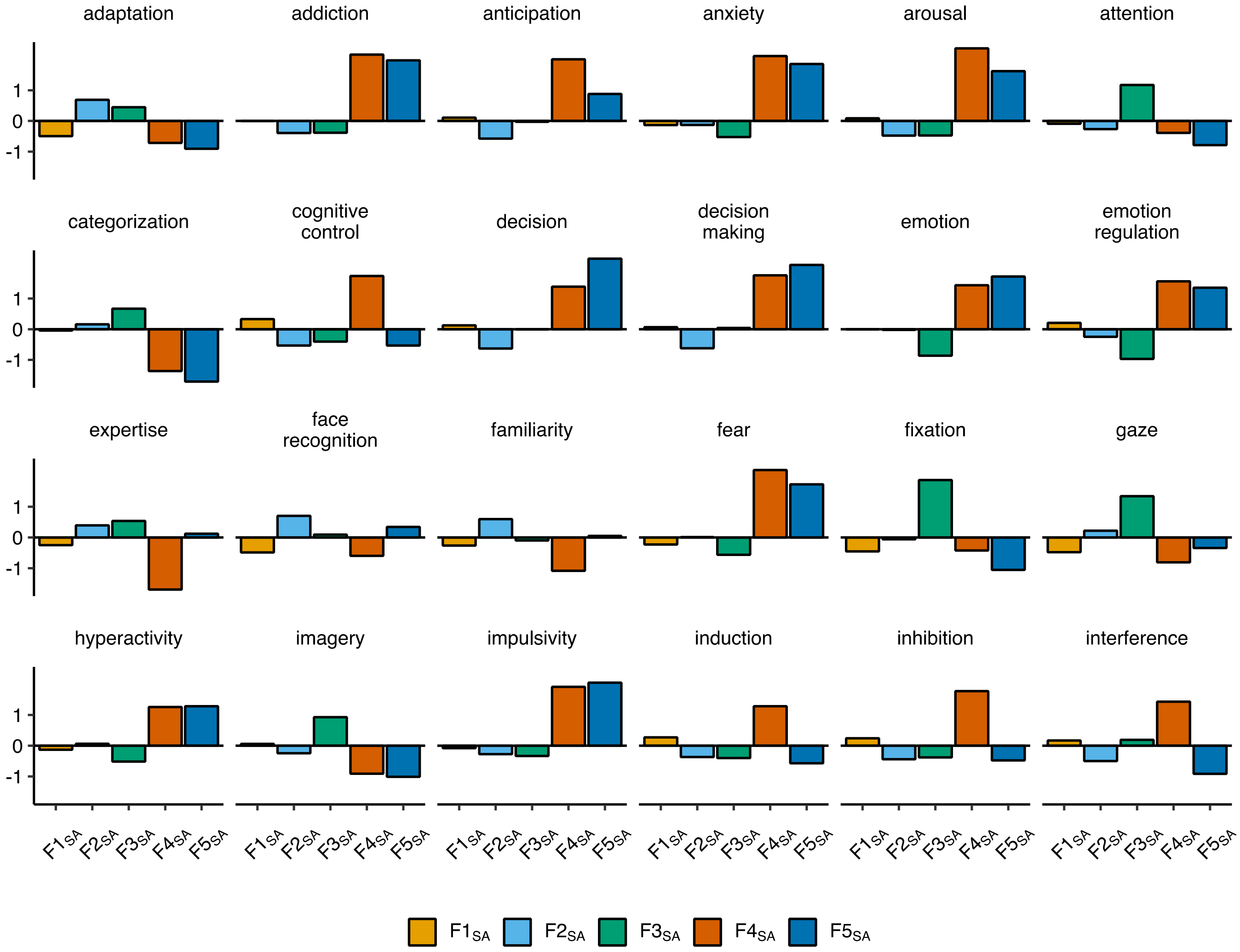

Supplementary Figure 10a. Functionally-derived features of the cortex that vary across the genomic factors of surface area (SA). Faceted bar charts of mean standardized values per factor for functionally-derived features of the cortex. All displayed features were significantly different across the five genomic factors of SA ($F1_{SA}$ to $F5_{SA}$) in an omnibus test of differences (FDR-corrected $P_{spin} < .05$; **Methods**). Full results are reported in Supplementary Table 9.

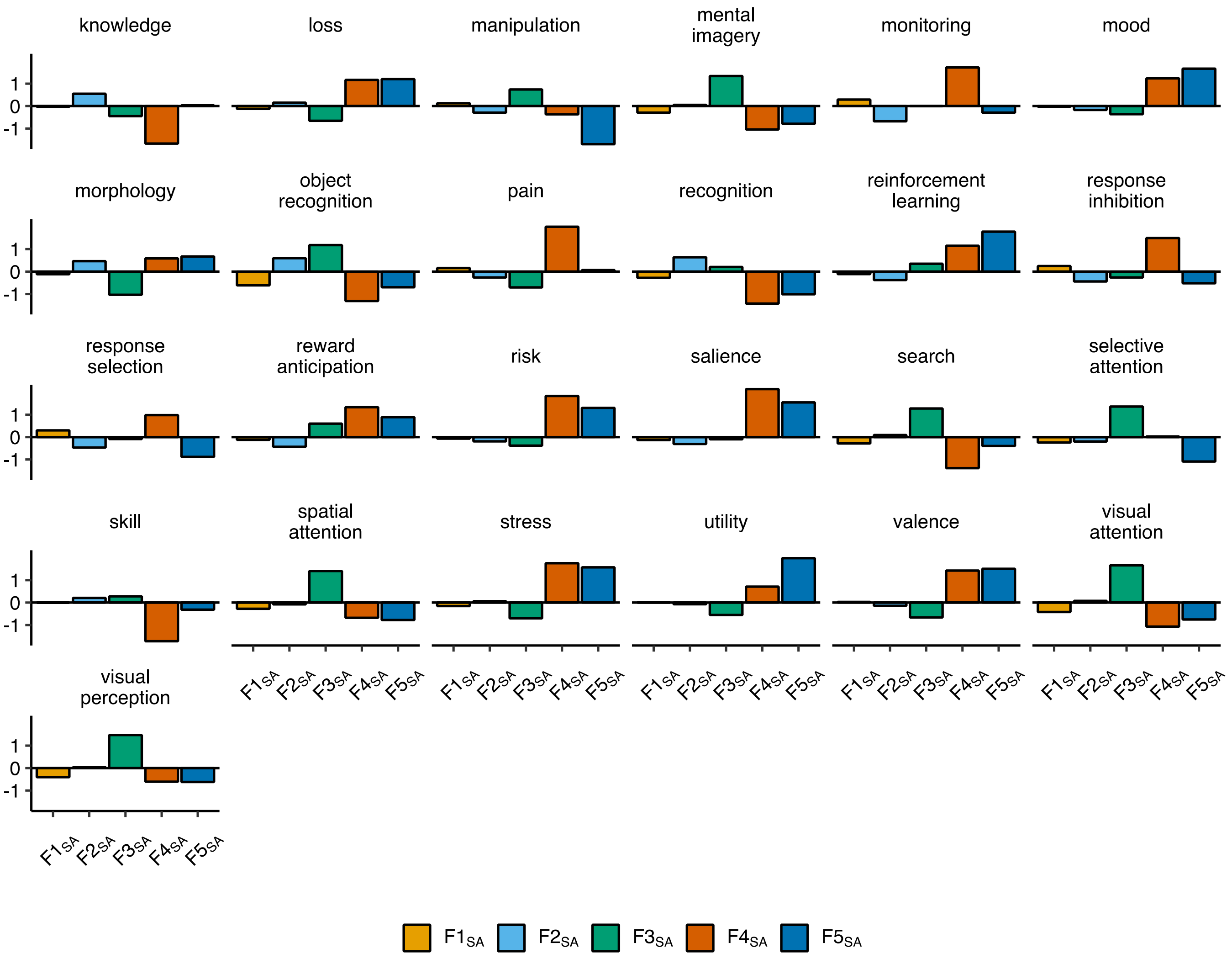

Supplementary Figure 10b. Functionally-derived features of the cortex that vary across the genomic factors of surface area (SA). Faceted bar charts of mean standardized values per factor for functionally-derived features of the cortex. All displayed features were significantly different across the five genomic factors of SA ($F1_{SA}$ to $F5_{SA}$) in an omnibus test of differences (FDR-corrected $P_{spin} < .05$; **Methods**). Full results are reported in Supplementary Table 9.

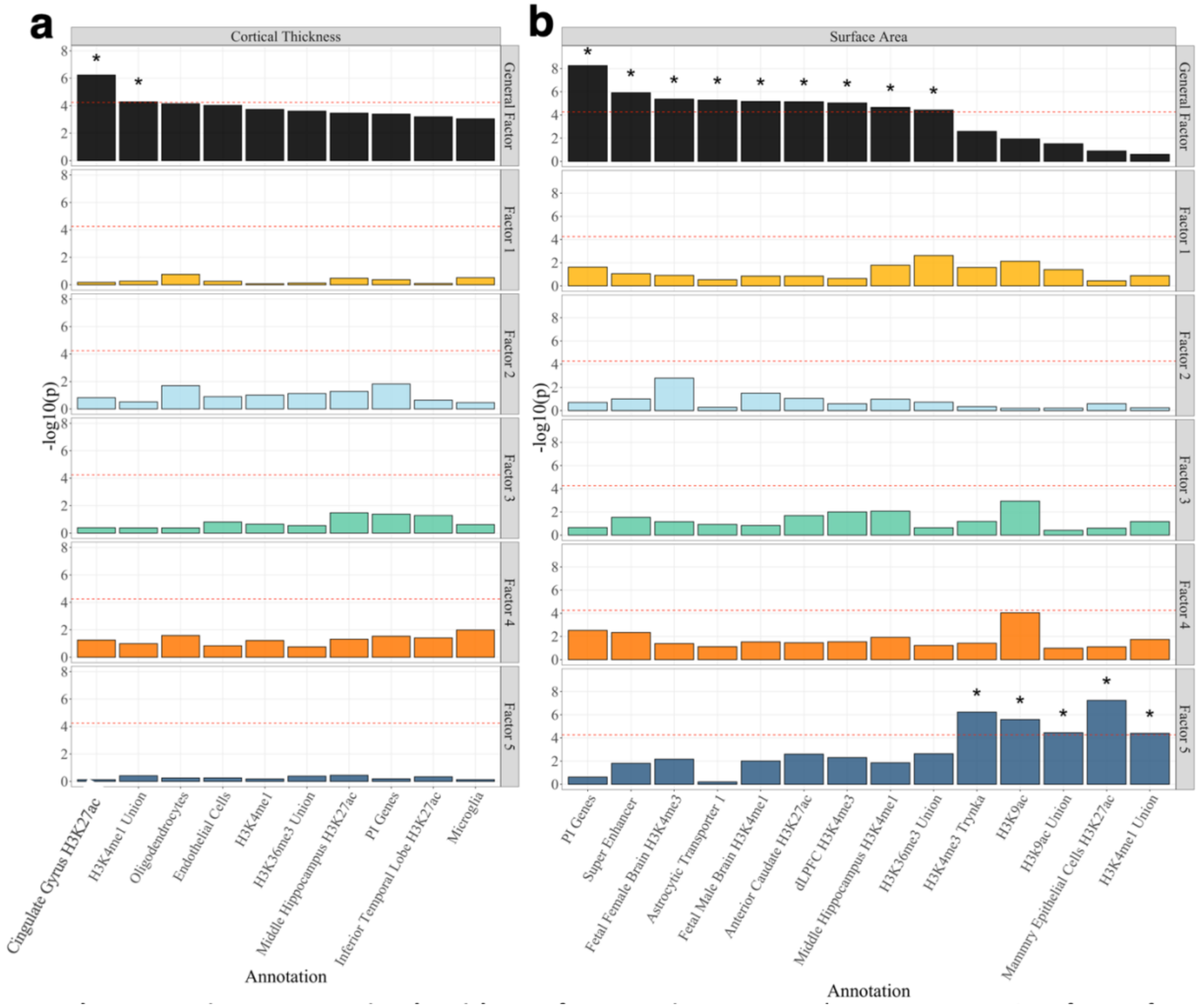

Supplementary Figure 11. Functional Enrichment for Genomic Factors in Bifactor Model. **a,** The top 10 annotations for any factor from the bifactor model for cortical thickness **b,** The 14 annotations that were Bonferroni significant from the bifactor model for surface area. In both panels, the rows depict the –log10(p) values for the enrichment estimates for the general factor at the top, followed by the five, residual factors from the bifactor model below. P-values are one-tailed and were calculated using the ratio of the enrichment estimate over its standard error. We correct for multiple testing for Stratified Genomic SEM results by employing a strict Bonferroni correction for the number of annotations (152) and factors (6) analyzed (i.e., p < 5.48E-5). Bars significant at a Bonferroni corrected threshold are depicted with a ***;** the red dashed line is the Bonferroni corrected threshold.

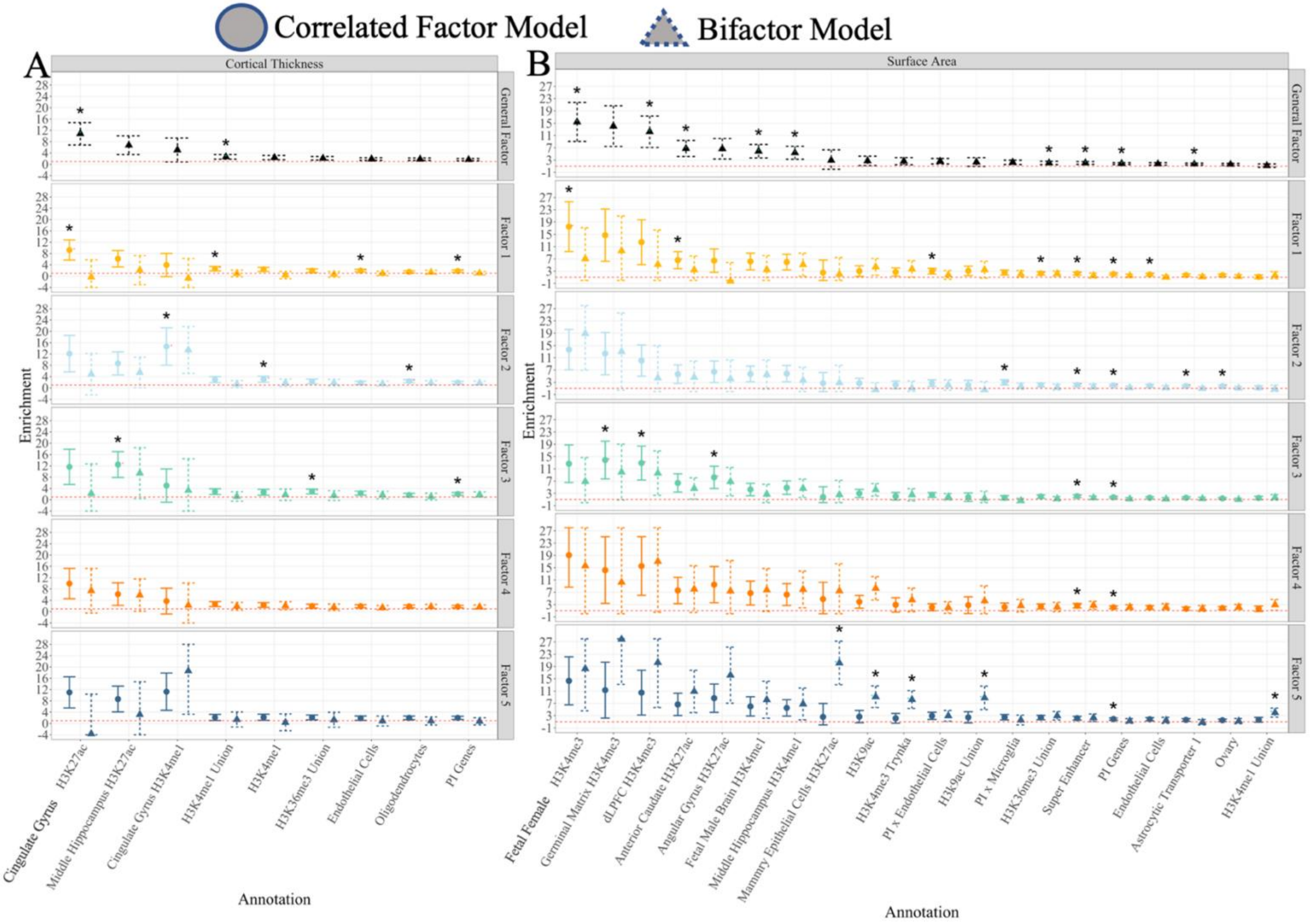

Supplementary Figure 12. Stratified Genomic SEM Results: Correlated and Bifactor Models. Figure depicts the 9 functional annotations for Cortical Thickness (*Panel **A***; N = 33,992) and 20 annotations for Surface Area (*Panel **B***; N = 33,992) that were significantly enriched at a Bonferroni corrected threshold for at least one of the factors for either the correlated factor or bifactor model results. The individual points reflect the enrichment estimate and the error bars around these points the 95% confidence intervals. These confidence intervals are capped at the y-axis limits for visualization purposes. P-values are one-tailed and were calculated using the ratio of the enrichment estimate over its standard error (see Supplementary Data 10 and 11 for exact p-values). We correct for multiple testing for Stratified Genomic SEM results by employing a strict Bonferroni correction for the number of annotations (152) and factors (6) analyzed (i.e., $p < 5.48E-5$). Annotations significant at a Bonferroni corrected threshold are depicted with a *. For Factors 1-5, results depicted in circles with solid error bars reflect results from the correlated factors model and triangles with dashed line error bars results from the bifactor model. Results for the general factor at the top of each panel are for the common factor defined by all 34 brain regions in the bifactor model. The horizontal red dashed line at 1 reflects the null of no enrichment.

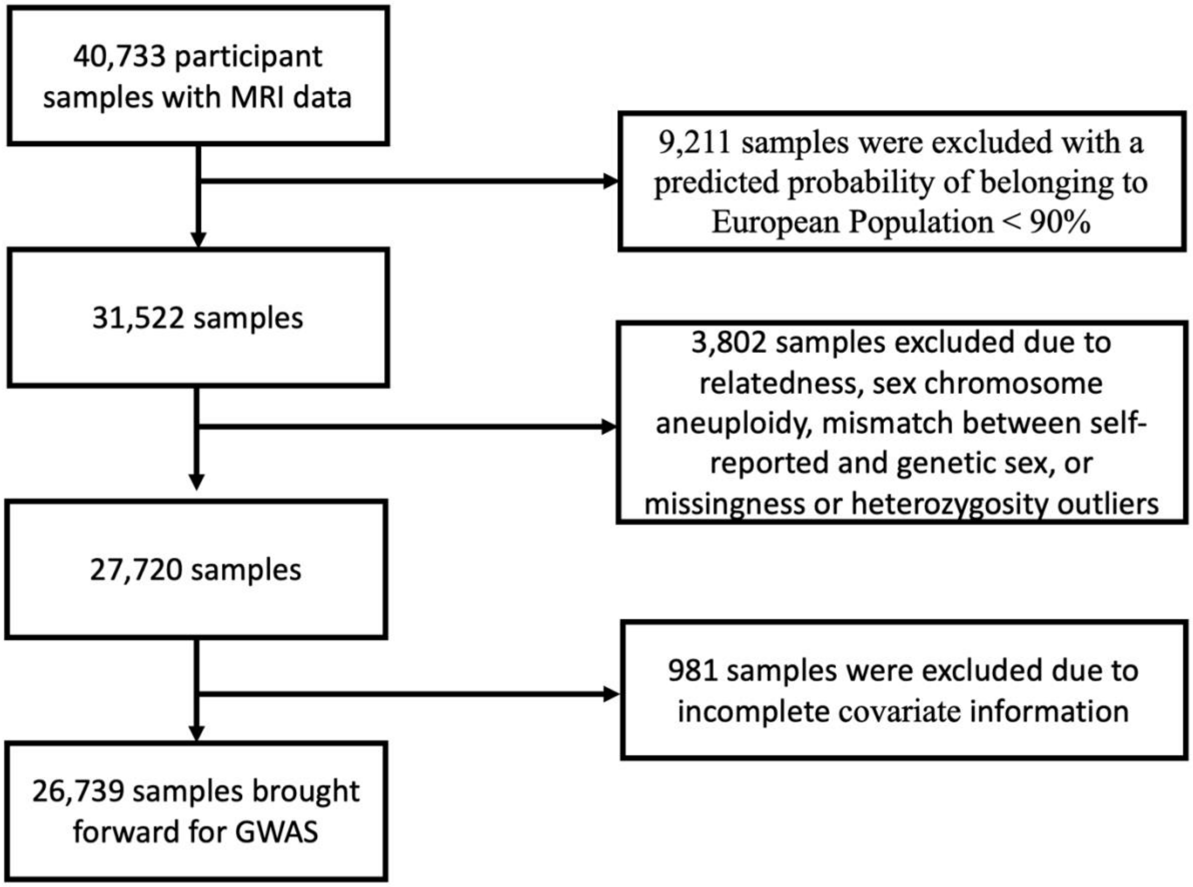

Supplementary Figure 13. UK Biobank Participant Sample Quality Control. Schematic depicts the series of filters, and participants removed at each step, used to perform quality control of the UKB data. Further details (e.g., how predicted probability of European ancestry was calculated) are provided in the **Methods.**

Supplementary References

1. Grasby, K. L. *et al.* The genetic architecture of the human cerebral cortex. *science* 367, (2020).

2. Ge, T. *et al.* The shared genetic basis of educational attainment and cerebral cortical morphology. *Cerebral Cortex* 29, 3471–3481 (2019).

3. Cauda, F. *et al.* Grey matter abnormality in autism spectrum disorder: an activation likelihood estimation meta-analysis study. *J Neurology Neurosurg Psychiatry* 82, 1304 (2011).

4. Chien, Y.-L., Chen, Y.-C. & Gau, S. S.-F. Altered cingulate structures and the associations with social awareness deficits and CNTNAP2 gene in autism spectrum disorder. *Neuroimage Clin* 31, 102729 (2021).
