# Supplementary material for: Multivariate genomic architecture of cortical thickness and surface area at multiple levels of analysis
Source: Nat Commun. 2023 Feb 20;14:946. doi: 10.1038/s41467-023-36605-x (PMC9941500; doi:10.1038/s41467-023-36605-x)
Supplement: Supplementary file 3 — Description of Additional Supplementary Files [file 41467_2023_36605_MOESM3_ESM.pdf]

Description of Additional Supplementary Files

File Name: Supplementary Data 1
Description: Exploratory Factor Analysis Standardized Loadings

File Name: Supplementary Data 2
Description: Cortical Thickness Genomic SEM Confirmatory Factor Model Results in ENIGMA

File Name: Supplementary Data 3
Description: Surface Area Genomic SEM Confirmatory Factor Model Results

File Name: Supplementary Data 4
Description: Model Fit from Confirmatory Factor Models fit in Genomic SEM

File Name: Supplementary Data 5
Description: Left Hemisphere Cortical Thickness Genomic SEM Confirmatory Factor Model Results in UK Biobank

File Name: Supplementary Data 6
Description: Right Hemisphere Cortical Thickness Genomic SEM Confirmatory Factor Model Results in UK Biobank

File Name: Supplementary Data 7
Description: Left Hemisphere Surface Area Genomic SEM Confirmatory Factor Model Results in UK Biobank

File Name: Supplementary Data 8
Description: Right Hemisphere Surface Area Genomic SEM Confirmatory Factor Model Results in UK Biobank

File Name: Supplementary Data 9
Description: Topographical Annotation

File Name: Supplementary Data 10
Description: Stratified Genomic SEM Enrichment Results for Cortical Thickness

File Name: Supplementary Data 11
Description: Stratified Genomic SEM Enrichment Results for Surface Area

File Name: Supplementary Data 12
Description: Genetics Correlations with the UKB g-factor

File Name: Supplementary Data 13
Description: Confirmatory Factor Results from Psychiatric Measurement Model

File Name: Supplementary Data 14
Description: Genetics Correlations with the Psychiatric Factors
