# Supplementary material for: Multivariate genomic architecture of cortical thickness and surface area at multiple levels of analysis
Source: Nat Commun. 2023 Feb 20;14:946. doi: 10.1038/s41467-023-36605-x (PMC9941500; doi:10.1038/s41467-023-36605-x)
Supplement: Supplementary file 5 — Reporting Summary [file 41467_2023_36605_MOESM5_ESM.pdf]

nature portfolio

Corresponding author(s): Andrew Grotzinger

Last updated by author(s): Jan 7, 2023

Reporting Summary

Nature Portfolio wishes to improve the reproducibility of the work that we publish. This form provides structure for consistency and transparency in reporting. For further information on Nature Portfolio policies, see our Editorial Policies and the Editorial Policy Checklist.

Statistics

For all statistical analyses, confirm that the following items are present in the figure legend, table legend, main text, or Methods section.

n/a	Confirmed	
☐	☒	The exact sample size (*n*) for each experimental group/condition, given as a discrete number and unit of measurement
☒	☐	A statement on whether measurements were taken from distinct samples or whether the same sample was measured repeatedly
☐	☒	The statistical test(s) used AND whether they are one- or two-sided<br>*Only common tests should be described solely by name; describe more complex techniques in the Methods section.*
☐	☒	A description of all covariates tested
☐	☒	A description of any assumptions or corrections, such as tests of normality and adjustment for multiple comparisons
☐	☒	A full description of the statistical parameters including central tendency (e.g. means) or other basic estimates (e.g. regression coefficient) AND variation (e.g. standard deviation) or associated estimates of uncertainty (e.g. confidence intervals)
☐	☒	For null hypothesis testing, the test statistic (e.g. *F*, *t*, *r*) with confidence intervals, effect sizes, degrees of freedom and *P* value noted<br>*Give P values as exact values whenever suitable.*
☒	☐	For Bayesian analysis, information on the choice of priors and Markov chain Monte Carlo settings
☒	☐	For hierarchical and complex designs, identification of the appropriate level for tests and full reporting of outcomes
☒	☐	Estimates of effect sizes (e.g. Cohen's *d*, Pearson's *r*), indicating how they were calculated

Our web collection on statistics for biologists contains articles on many of the points above.

Software and code

Policy information about availability of computer code

Data collection	No software was used for data collection.
Data analysis	Genetic correlations, factor modeling, associations with external traits, and enrichment analyses were conducted using the Genomic SEM package available for download at https://github.com/GenomicSEM/GenomicSEM. Genomic SEM (and the Stratified Genomic SEM extension) were run using version 0.0.5 and implemented using R version 3.5.1. Exploratory factor analyses were conducted using the factanal R package. GWAS run in the UKB sample were run using plink version 2.0.<br>The figures that overlay the current findings over images of the human brain (e.g., Figure 1c) were created using the BrainsForPublication software:<br>https://github.com/WhitakerLab/BrainsForPublication

For manuscripts utilizing custom algorithms or software that are central to the research but not yet described in published literature, software must be made available to editors and reviewers. We strongly encourage code deposition in a community repository (e.g. GitHub). See the Nature Portfolio guidelines for submitting code & software for further information.

Data

Policy information about availability of data

All manuscripts must include a data availability statement. This statement should provide the following information, where applicable:

- Accession codes, unique identifiers, or web links for publicly available datasets
- A description of any restrictions on data availability
- For clinical datasets or third party data, please ensure that the statement adheres to our policy

Data for the UK Biobank (UKB) can be requested from:
https://bbams.ndph.ox.ac.uk/ams/signup
Cortical maps of intracortical microstructure, laminar differentiation, cellular/neuronal density were generated using data from the BigBrain project at:
https://bigbrainproject.org/maps-and-models.html
Cortical maps of neurotransmitter receptor and transporter densities can be obtained from:
https://github.com/netneurolab/hansen_receptors
Probabilistic association cortical maps of 123 cognitive and psychological processes (generated using data from Neursynth [https://neurosynth.org/]) can be obtained from:
https://github.com/netneurolab/hansen_receptors.
Cortical maps of cell-type specific transcriptional signatures were generated using data from the Allen Human Brain Atlas at:
https://human.brain-map.org/static/download

Human research participants

Policy information about studies involving human research participants and Sex and Gender in Research.

Reporting on sex and gender	The breakdown of the ENIGMA participant sample by biological sex (determined from genotype data) was 51.6% female. Biological sex and age x sex interactions were included as covariates when running the GWAS analyses. The biological sex (determined from genotype data) of the UK Biobank sample is 54.1% female. Biological sex was included as a covariate when running GWAS analyses. Results are not split into sex-specific analyses due to the reduction in power that would come from halving the sample size within ENIGMA or the UKB.
Population characteristics	The age range in the ENIGMA sample across the 50 contributing European cohorts is 3-91. These cohorts reflect a mixture of population based and case-control samples. Case-control samples within ENIGMA included studies on schizophrenia, major depressive disorder, bipolar disorder, Parkinson's disease, anxiety, and epilepsy. The meta-analytic ENIGMA sample was reported to consist of 87.5% healthy participants (including the population-based samples). The UK Biobank is a population-based sample ages 40-69.
Recruitment	As there were 50 participant cohorts, each with their own recruitment protocols, we refer to the original ENIGMA paper (Grasby et al., 2020) of the 34 cortical brain regions for further details. For the UK Biobank replication sample, participants were recruited using a population-based study design among individuals living in the UK ages 40-69 with an ~5% response rate; this low response rate may bias results based on participant characteristics that self-selected into the UKB study, though it is not possible in the current analyses to quantify the degree or nature of this bias. As with any analyses using the UKB participant sample, results should be interpreted accordingly.
Ethics oversight	Each of the individual 50 cohorts that participated in ENIGMA received approval from the relevant oversight group. As one example, the ALSPAC cohort received approval from the ALSPAC Ethics and Law Committee and the Local Research Ethics Committees. The UKB replication sample received ethical approval from the North West Centre Research Ethics Committee (REC number 11/NW/0382). The current analyses were conducted under the approved UKB application 32568.

Note that full information on the approval of the study protocol must also be provided in the manuscript.

Field-specific reporting

Please select the one below that is the best fit for your research. If you are not sure, read the appropriate sections before making your selection.

☐ Life sciences ☒ Behavioural & social sciences ☐ Ecological, evolutionary & environmental sciences

For a reference copy of the document with all sections, see nature.com/documents/nr-reporting-summary-flat.pdf

Behavioural & social sciences study design

All studies must disclose on these points even when the disclosure is negative.

Study description	Our study uses a quantitative design wherein we utilize GWAS summary statistics to examine the multivariate architecture of 34 brain regions for cortical thickness and surface. We specifically utilize the publicly available GWAS summary statistics from the

	ENIGMA consortium. These summary statistics reflect the meta-analytic estimates across a range of contributing cohorts, with a total sample size of 33,992 and an participant age range of 3-91. At the genome-wide level, we apply Genomic SEM to identify 5 correlated factors and examine the relationships between these factors and relevant external traits (e.g., cognitive and psychiatric traits). Using a novel method, Stratified Genomic SEM, we go on to examine multivariate enrichment at the level of the brain factors. Finally, we conduct a series of spin-based tests to examine the relationship between the factor structure we identify map onto previously describe biological and functional partitionings of the cortex.
Research sample	We use publically available summary statistics (links and details provided in the data availability statement and method section of the manuscript) for cortical indices, psychiatric traits, and cognitive traits. As Genomic SEM relies on ld-score regression (LDSC) to construct genetic covariance matrices, and LDSC requires summary statistics to be within a single ethnic population due to differences in linkage disequilibrium across populations, we use only summary statistics restricted to European populations. Therefore, our findings are not representative beyond European populations, and as we highlight in the manuscript it will be critical for future work to re-examine these research questions for different ancestry groups.
Sampling strategy	As we use previously collected GWAS data from outside groups, and did not collect any participant samples ourselves, this is not applicable. We did not use statistical methods to predetermine sample size as the project aim was to use the largest GWAS samples available, which are themselves taken to be well-powered. Briefly, we note that the results utilized from the ENIGMA consortium reflects a meta-analysis from 50 different European ancestry cohorts that largely employed a population based or case-control study design. The UK Biobank sample (used for our replication analyses) is a population based sample that recruited individuals ages 40-69 living in the United Kingdom.
Data collection	This is not applicable as we use previously collected GWAS data that we were not involved in collecting. We note briefly here that within ENIGMA the imaging data was collected using T1-weighted magnetic resonance imaging (MRI) scans and that genotype data was imputed to either 1000 Genomes or the Haplotype Reference Consortium (HRC).
Timing	This is not applicable as we use previously collected data. We began curating GWAS summary statistics in January 2021 and put a freeze on including any new GWAS datasets on March 2021.
Data exclusions	We use only summary statistics from European populations due to the requirements of LDSC, as noted above. Within ENIGMA, each of the 60 cohorts are reported to have visually inspected the imaging data to exclude failed segmentations. Using pre-established quality control criteria, individual regions > 2.698 SD from the sample mean were specifically examined to determine whether they should be excluded from analysis. As each of the 50 European sample, participating cohorts applied exclusion criteria based on individual brain regions passing quality control, there is not a single number that describes the number of participants removed from the ENIGMA analyses. However, the majority of cohorts can be said to have excluded < 5% of data for each of the 34 brain regions.
Non-participation	This is not applicable as we use previously collected data. Details on levels of participation can be found in the primary ENIGMA publication examining the genetic underpinnings of the 34 cortical regions.
Randomization	This is not applicable as there were no experimental groups in the ENIGMA or the UK Biobank imaging samples.

Reporting for specific materials, systems and methods

We require information from authors about some types of materials, experimental systems and methods used in many studies. Here, indicate whether each material, system or method listed is relevant to your study. If you are not sure if a list item applies to your research, read the appropriate section before selecting a response.

Materials & experimental systems

n/a	Involved in the study	
☒	☐	Antibodies
☒	☐	Eukaryotic cell lines
☒	☐	Palaeontology and archaeology
☒	☐	Animals and other organisms
☒	☐	Clinical data
☒	☐	Dual use research of concern

Methods

n/a	Involved in the study	
☒	☐	ChIP-seq
☒	☐	Flow cytometry
☒	☐	MRI-based neuroimaging
